# Improved virus-induced gene silencing allows discovery of a serpentine synthase gene in *Catharanthus roseus*

**DOI:** 10.1093/plphys/kiab285

**Published:** 2021-06-19

**Authors:** Kotaro Yamamoto, Dagny Grzech, Konstantinos Koudounas, Emily Amor Stander, Lorenzo Caputi, Tetsuro Mimura, Vincent Courdavault, Sarah E. O’Connor

**Affiliations:** 1 Department of Natural Product Biosynthesis, Max Planck Institute for Chemical Ecology, Jena 07745, Germany; 2 Graduate School of Pharmaceutical Sciences, Chiba University, Chiba 263-8522, Japan; 3 EA2106 “Biomolécules et Biotechnologies Végétales”, Université de Tours, Tours 37200, France; 4 Department of Biology, Graduate School of Science, Kobe University, Kobe, Hyogo 657-8501, Japan

## Abstract

Specialized metabolites are chemically complex small molecules with a myriad of biological functions. To investigate plant-specialized metabolite biosynthesis more effectively, we developed an improved method for virus-induced gene silencing (VIGS). We designed a plasmid that incorporates fragments of both the target gene and knockdown marker gene (phytoene desaturase, PDS), which identifies tissues that have been successfully silenced in planta. To demonstrate the utility of this method, we used the terpenoid indole alkaloid (TIA) pathway in Madagascar periwinkle (*Catharanthus roseus*) as a model system. *Catharanthus roseus* is a medicinal plant well known for producing many bioactive compounds, such as vinblastine and vincristine. Our VIGS method enabled the discovery of a previously unknown biosynthetic enzyme, serpentine synthase (SS). This enzyme is a cytochrome P450 (CYP) that produces the β-carboline alkaloids serpentine and alstonine, compounds with strong blue autofluorescence and potential pharmacological activity. The discovery of this enzyme highlights the complexity of TIA biosynthesis and demonstrates the utility of this improved VIGS method for discovering unidentified metabolic enzymes in plants.

## Teaser

An improved virus-induced gene silencing approach led to the discovery of the alkaloid biosynthetic enzyme serpentine synthase.

## Introduction

To understand how plants coordinate production of metabolites, the function of biosynthetic genes, regulatory elements, and transporters must be assessed in planta. Virus-induced gene silencing (VIGS) is a widely used method in which genes can be silenced in planta in a relatively rapid time frame (Courdavault et al., 2020). In a typical VIGS experiment, the chemotype of the gene-silenced plant is qualitatively and quantitatively analyzed, allowing functional characterization of enzyme activity without access to purified substrates, and also confirming whether the enzyme activity is physiologically relevant in vivo. Although VIGS is an indisputably powerful method for discovering and characterizing biosynthetic genes, the results of VIGS are often hampered by lack of sensitivity, lack of reproducibility, and high error among biological replicates. Here we developed a simple modification of a widely used VIGS system (Liscombe and O’Connor, 2011) to address these problems.

**Figure 1:**
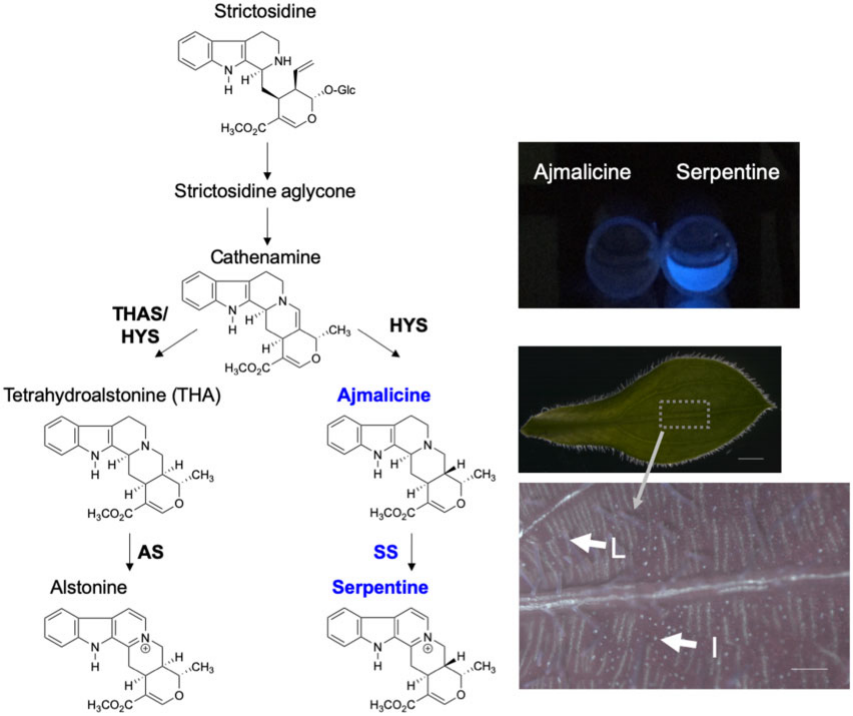
Serpentine biosynthesis in planta. The branch of the TIA biosynthesis pathway in *C. roseus* leading to serpentine and alstonine is shown. Alstonine is made by reduction of cathenamine to THA, followed by oxidation of THA to alstonine by the previously reported enzyme AS. The diastereomer of alstonine, serpentine, is made via a biochemically similar pathway. Cathenamine is reduced to the diasteomer ajmalicine, which is then oxidized to serpentine. The enzyme catalyzing this last oxidative step of serpentine biosynthesis was unknown at the outset of this study. Serpentine is fluorescent, while the precursor ajmalicine is not, which has led to the identification of idioblast (I) and laticifer (L) cells on *C. roseus* leaves that accumulate serpentine (Yamamoto et al., 2016, 2019). Scale bar (Bright field image) = 1 mm, Scale bar (Fluorescent image) = 200 μm.

The terpenoid indole alkaloid (TIA) pathway, which produces thousands of alkaloids, many with potent biological functions and pharmaceutical properties (O'Connor and Maresh, 2006; El-Sayed and Verpoorte, 2007; Stöckigt et al., 2007; Facchini and De Luca, 2008; Dugé de Bernonville et al., 2015), is an excellent system for testing an improved VIGS method. *Catharanthus roseus* (L.) G. Don (Apocynaceae) is one of the best-characterized TIA producing plants, producing the commercially valuable compounds vinblastine and vincristine, which are used as anti-cancer drugs (Van der Heijden et al., 2004; Gigant et al., 2005; Kavallaris, 2010). Extensive studies have revealed that *C. roseus* produces over 100 TIAs from strictosidine, which is the central precursor for all TIAs (Figure 1; Van der Heijden et al., 2004). However, despite extensive study, many TIA biosynthetic genes in *C. roseus* remain unelucidated.

**Figure 2:**
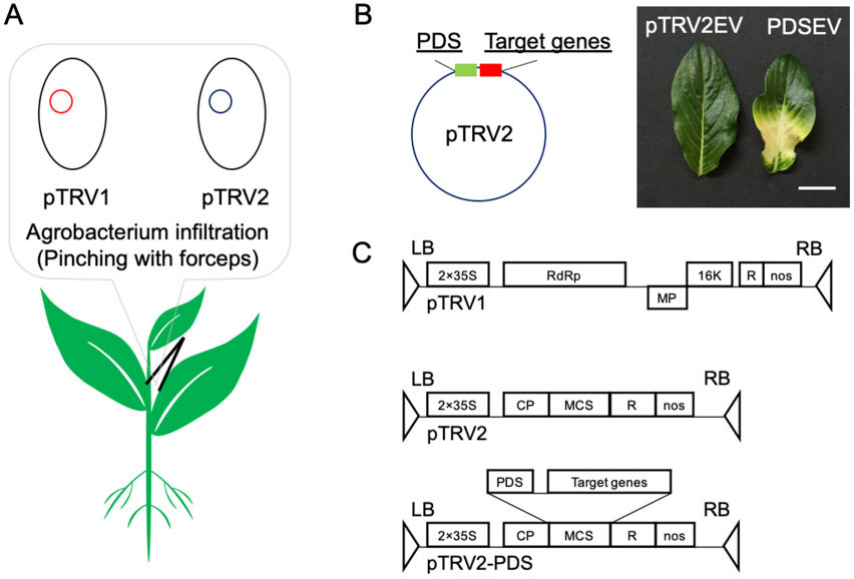
Improved VIGS method for screening of gene function. A, General VIGS method with pTRV1 (red) and pTRV2 (blue) vectors. B, Schematic of VIGS constructs for double knockdown with marker gene (PDS; PDS+ EV, PDSEV). The bleaching phenotype resulting from silencing of PDS is shown. Scale bar shows 1 cm. C, Design of pTRV1 and pTRV2 vectors. LB, left border; RB, right border; 35S, 35S promoter; RdRp, RNA-dependent RNA polymerase; MP, movement protein; 16K, 16-kDa cysteine-rich protein; R, self-cleaving ribozyme; nos, nos terminator; CP, coat protein; MCS, multiple cloning site.

The tobacco rattle virus-based VIGS platform has been an important and widely used tool for screening of gene function in many plants (Figure 2A; Liu et al., 2002; Burch-Smith et al., 2004, 2006; Liscombe and O’Connor, 2011). However, in many plants, including *C. roseus*, gene knockdown with the pTRV2 system happens sporadically and fails to affect the entire plant—or even the entire organ—which was subjected to infection (Supplemental Figure S1). In this case, the analysis of VIGS experiments must take into account many partially silenced samples, which obscure functional characterization of genes. Here, we report a modified VIGS construct that harbors the gene of interest along with a marker gene, phytoene desaturase (PDS; Figure 2, B and C). Through observation of leaf photobleaching, this double knockdown system allows pinpointing of the exact tissues that have been subjected to successful VIGS-mediated gene knockdown, hence facilitating the study of tissue-specific metabolism, and reduces the experimental time and required replicates. We provide proof of concept for the efficacy of this improved VIGS method by dissecting the final step of the heteroyohimbine pathway that is responsible for alstonine and serpentine production in *C. roseus* leaf tissue.

## Results

### Heteroyohimbine-type TIAs in *C. roseus*

The TIAs serpentine and alstonine, known as heteroyohimbine type TIAs, are produced in two steps from two distinct stereoisomers downstream of the deglycosylated central intermediate strictosidine (Figure 1; El-Sayed and Verpoorte, 2007). Serpentine inhibits topoisomerase, while alstonine has reported anti-psychotic activity (Dassonneville et al., 1999; Elisabetsky and Costa-Campos, 2006). Interestingly, serpentine has beautiful blue autofluorescence when excited with ultraviolet light (UV) (Hisiger and Jolicoeur, 2007), and this has facilitated the isolation of idioblast and laticifer cells in *C. roseus*, the cell types in which this compound accumulates, for single cell metabolomics (Figure 1; Yamamoto et al., 2016, 2019).

It was previously reported that several medium chain alcohol dehydrogenases reduce an isomer of deglycosylated strictosidine, cathenamine, to tetrahydroalstonine (THA; THA synthase), or to a mixture of THA and ajmalicine (heteroyohimbine synthase; Stavrinides et al., 2015, 2016). Although early studies suggested that the tetrahydro-β-carboline moieties of ajmalicine and THA are oxidized by a peroxidase localized in the vacuoles to yield serpentine and alstonine, respectively (Blom et al., 1991), one cytochrome P450 (alstonine synthase, AS) converted THA to alstonine in vitro (Dang et al., 2018). However, AS is not expressed in leaf (only in stem and root) although the β-carboline alkaloid serpentine is observed in leaf tissue (Supplemental Figure S2). In addition, the catalytic efficiency of the conversion of ajmalicine to serpentine by AS is low (Dang et al., 2018). Thus, it is likely that another enzyme with greater specificity for conversion of ajmalicine to serpentine, as well as leaf-specific localization, exists in addition to AS in planta. We chose identification of the missing “serpentine synthase” (SS) as a target on which to test an improved VIGS system.

### A dual VIGS-construct for improved resolution

The reported VIGS methodology for *C. roseus* (Liscombe and O’Connor, 2011) assumes that the two youngest leaves that emerge postinfection will be silenced, and data acquired with this method simply harvests these leaves without verifying the location of silencing. We reasoned that our analyses could be improved by modifying the VIGS method such that we could easily visualize the tissues, or specific leaves, which have been silenced. This would greatly improve the sensitivity and statistical reproducibility of such VIGS analyses, particularly for tissues in which there are low or variable levels of a metabolite.

To track which leaves of *C. roseus* were specifically silenced, we designed a pTRV2 construct that incorporated PDS as a marker of successful silencing (Figure 2, B and C). PDS is involved in the biosynthesis of the photoprotective carotenoids, and knockdown of phytoene desaturation causes destruction of chlorophyll by photooxidation (Senthil-Kumar and Mysore, 2014). Since PDS knockdown causes obvious bleaching of the chlorophyll-containing tissues, this gene has been used as a VIGS marker for checking the optimal time to harvest silenced tissue, but the strategy of using a double knockdown to pinpoint the location of silencing has never been reported (Carqueijeiro et al., 2015). The PDS marker can be visualized in any tissue that has chlorophyll, namely leaf, stem, and fruit.

Catharanthine synthase (CS), a gene involved in biosynthesis of TIA catharanthine that is highly expressed in leaf, was subjected to knockdown using this vector system as a positive control. We measured the metabolites of bleached leaves with LC–MS, which indicated that levels of catharanthine were reduced, as previously reported (Caputi et al., 2018; Qu et al., 2018; Supplemental Figures S3, S4). Therefore, we concluded that this pTRV2-PDS vector is well-suited for gene discovery in *C. roseus* leaf tissue.

### Use of the improved dual vector to identify SS

We used the recently reported AS (Dang et al., 2018), a cytochrome P450, as a query for a BLAST analysis of the *C. roseus* transcriptome to search for SS candidate genes (Supplemental Figure S5). The retrieved genes were then organized into a phylogenetic tree, and three homologs were selected (CRO_T111318, CRO_T135961, and CRO_T127473) based on similarity to AS.

We conducted knockdown analysis with pTRV2EV (empty vector [EV]), PDSEV (PDS marker only), and PDS-target gene (PDS marker plus gene of interest) in 4-weeks-old *C. roseus* plants, using the PDS-target gene vector for the three selected candidate genes. Four-week-old *C. roseus* plants were infected with *Agrobacterium tumefaciens* containing the constructs of interest, and then grown for an additional 4 to 5 weeks. Only leaves showing the bleached phenotype indicative of successful silencing were selected for analysis. The alkaloids were extracted with MeOH containing an internal standard (2 ppm ajmaline). The amount of MeOH for extraction was calculated and normalized with the fresh weight of each sample. The alkaloid content was then measured with LC–MS.

**Figure 3:**
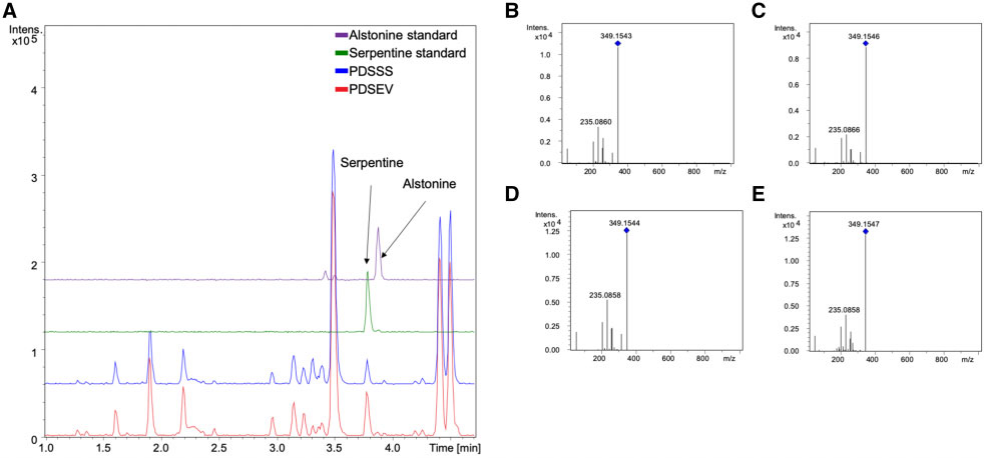
LC–MS analysis of *C. roseus* leaves with silenced serpentine synthase: levels of the expected product serpentine decrease. A, Presence of alstonine and serpentine metabolites in extracts of plants subjected to VIGS. Only the serpentine stereoisomer is observed, and a decrease in the level of this metabolite can be observed in silenced plants (PDSSS). B, MS2 fragments of *m*/*z* 349.15 in PDSEV sample. C, MS2 fragments of *m/z* 349.15 in PDSSS sample. D, Serpentine standard. E, Alstonine standard. Blue diamonds indicate parent ion. For all panels, *x*-axis is retention time in minutes, while *y*-axis is ion intensity in arbitrary units.

**Figure 4:**
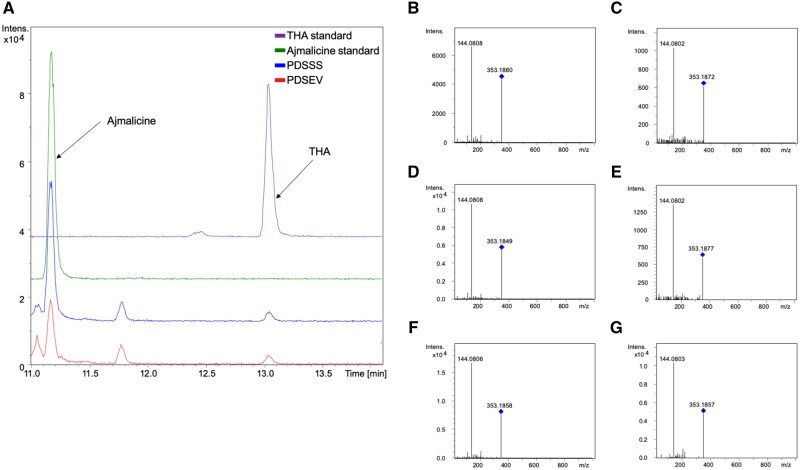
LC–MS analysis of *C. roseus* leaves with silenced serpentine synthase: levels of the substrate ajmalicine increase. Separation of ajmalicine and THA requires an extended LC method. A, The presence of these metabolites in extracts of plants subjected to VIGS. Much higher levels of ajmalicine compared with THA are observed in leaves. Ajmalicine levels increase after silencing of CrSS (PDSSS). B, MS2 fragments of *m*/*z* 353.18 (retention time = 11.16) in PDSEV sample. C, MS2 fragments of *m*/*z* 353.18 (rt = 13.02) in PDSEV sample. D, MS2 fragments of *m*/*z* 353.18 (rt = 11.17) in PDSSS sample. E, MS2 fragments of *m*/*z* 353.18 (rt = 13.03) in PDSSS sample. F, Ajmalicine standard (rt = 11.17). G, THA standard (rt = 13.03). Blue diamonds indicate parent ion. For all panels, *x*-axis is retention time in minutes, while *y*-axis is ion intensity in arbitrary units.

The knockdown of PDS_CRO_T111318 (PDS and CRO_T111318, subsequently named PDSSS for SS) showed a decrease in accumulation of a compound with *m*/*z* 349.15, which corresponds to the pseudomolecular ion [M]^+^ of serpentine and alstonine (Figure 1; Supplemental Figure S3). The retention time corresponded to serpentine, the expected product for SS (Figure 3; Supplemental Figure S6). In addition, a compound with *m*/*z* 353.18 was shown to accumulate upon silencing. The mass of this compound corresponds to the pseudomolecular ion [M + H]^+^ of ajmalicine and THA, with ajmalicine being the expected substrate for SS (Supplemental Figure S6). Ajmalicine and THA can only be separated using an extended LC method; this method validated that it was the ajmalicine isomer that increased upon silencing (Figure 4). The efficiency of knockdown for PDSSS and the positive control PDSCS was confirmed with RNA-Seq analysis (Supplemental Figure S4). The other two CYP candidates that were selected, CRO_T135961 and CRO_T127473, did not show any difference in alkaloid chemotype after silencing by this VIGS method and were not considered further.

On this basis, we concluded that CRO_T111318 was likely the enzyme that converts one or both of these alkaloids to their downstream products—serpentine and alstonine, respectively—in leaves, and was thus renamed SS, or CrSS. 

### Functional characterization of CrSS

**Figure 5:**
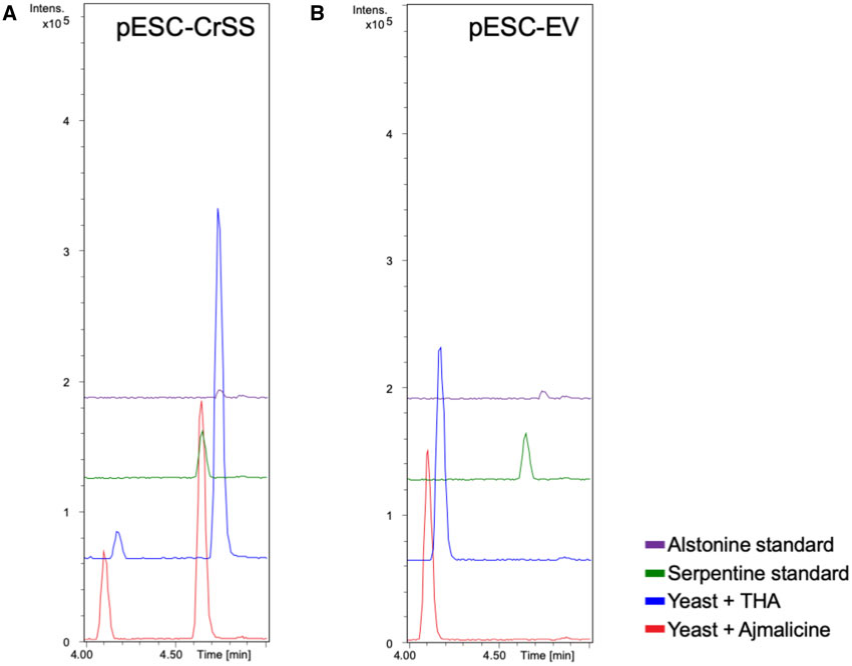
CrSS whole cell assay. *Saccharomyces cerevisiae* yeast cultures expressing CrSS incubated with either ajmalicine or THA. A, Incubation of yeast expressing CrSS with ajmalicine or THA. B, pESC-EV incubated with ajmalicine or THA. For all panels, *x*-axis is retention time in minutes, while *y*-axis is ion intensity in arbitrary units.

We heterologously expressed CrSS in baker’s yeast (*Saccharomyces cerevisiae)* to validate the activity of this enzyme in vitro. Consistent with the VIGS data, CrSS produced serpentine from ajmalicine when incubated in the media of yeast cultures expressing CrSS (Figure 5). In addition, this cytochrome P450 could also produce alstonine from THA (Figure 5). When microsomal fractions from yeast transformed with CrSS were challenged in a competition assay with both THA and ajmalicine, CrSS microsomes produced both oxidized products (Supplemental Figure S7 and S8). This substrate specificity is different to that of the previously discovered AS, which only turns over trace amounts of ajmalicine to form serpentine (0.6% of relative conversion of ajmalicine normalized to 100% conversion of THA; Dang et al., 2018). In addition, the gene expression profile of these genes showed that CrSS is expressed in leaf tissues, in contrast to CrAS, which is mainly expressed in stem and root (Supplemental Figure S2A). Notably, the expression pattern of CrSS is comparable to the metabolic profile of both ajmalicine (the preferred substrate of CrSS) and serpentine (Supplemental Figure S2, B and C).

We additionally asked whether a transient overexpression assay of CrSS would also result in an increase in serpentine in planta. Analysis of serpentine content in *C. roseus* leaves agroinfiltrated with the pEAQ-HT:CrSS-6HIS construct revealed that serpentine increased by 15.6% compared to leaves agroinfiltrated with an EV (Supplemental Figure S9 and S10). Successful overexpression of CrSS was further confirmed by Western blot allowing the detection of a single band of ∼59 kDa (Supplemental Figure S10). Therefore, overexpression assays further confirm that the serpentine content in leaves of *C. roseus* is directly related to the abundance of CrSS transcripts.

To ascertain the subcellular localization of CrSS, we first performed an in silico prediction of subcellular localization. Two independent servers suggested that CrSS is localized in the endoplasmic reticulum (ER; DeepLoc predicted a likelihood of 96.16%; LocTree3 gave a score of 32 with an expected accuracy of 86%) In addition, the TMHMM server identified a transmembrane region at aa 7–24 (FWSLPAIALLQVFLFFLF). To experimentally validate this prediction, we generated a YFP-fused construct of CrSS at the C-terminal maintaining the functionality of the predicted N-terminal transmembrane helix. Epifluorescence microscopy of *C. roseus* cells transiently expressing the CrSS-YFP construct revealed that CrSS perfectly colocalized with the ER-CFP marker, thus confirming that CrSS is localized in the ER (Supplemental Figure S11).

### Comparison of standard and modified VIGS methods

**Figure 6:**
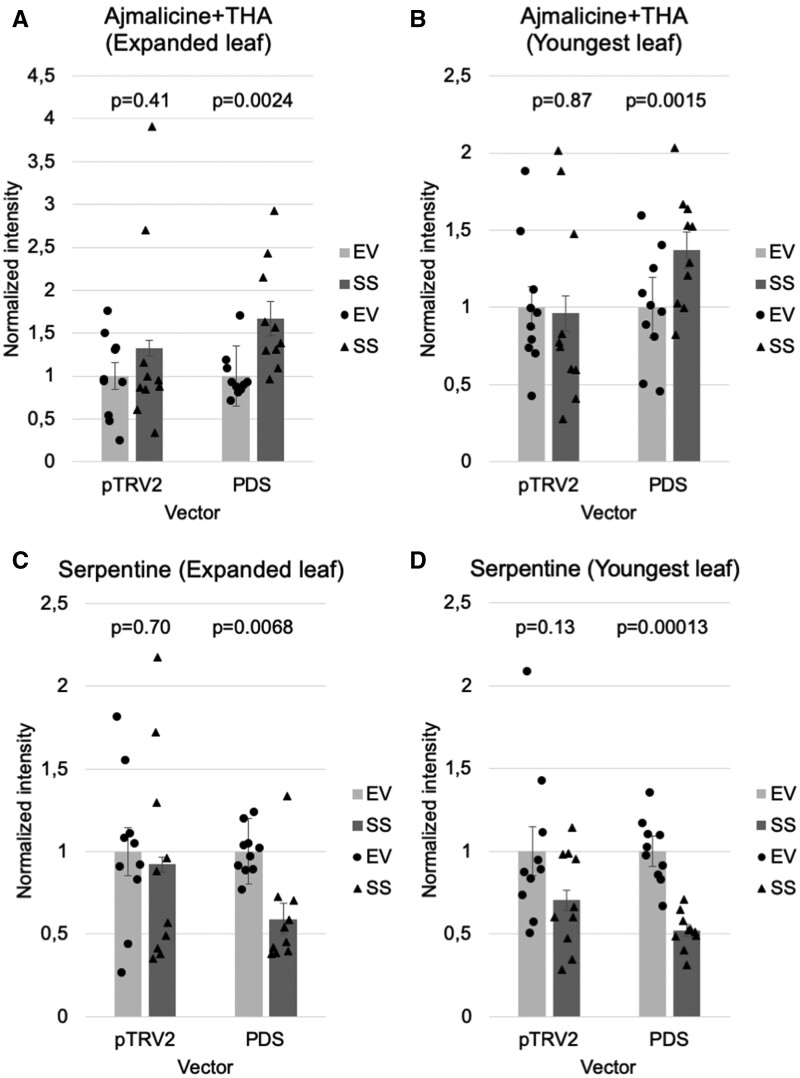
Comparison of standard (pTRV2) and modified (PDS) VIGS method. A, Levels of ajmalicine/THA (substrate; *m*/*z* 353.18) measured in expanded leaf pairs (third youngest leaf). B, Levels of ajmalicine/THA (substrate; *m*/*z* 353.18) measured in the youngest leaf pairs. C, Levels of serpentine (product; *m*/*z* 349.15) measured in expanded leaf pairs (third youngest leaf). D, Levels of serpentine (product; *m*/*z* 349.15) measured in the youngest leaf pairs. A–D, Only bleached leaves were selected for the pTRV2-PDS data. The intensity level was calculated and normalized (with the control value normalized as 1) with the LC–MS data from knockdown samples (*n* = 10). The amount of MeOH for extraction was calculated and normalized with the fresh weight of each sample for making the LC–MS chromatograms. Ajmaline was used as an internal standard. Error bars show se. Student *t* test was used for statistical analysis and *P*-value is shown above the data for each EV/SS vector pair.

Finally, we aimed to show conclusively that our silencing method provides a higher level of sensitivity compared to the standard VIGS method performed without a marker. To do this, we directly compared levels of both ajmalicine (CrSS substrate) and serpentine (CrSS product) in leaves where CrSS had been silenced, using either pTRV2 or the vector reported here, pTRV2-PDS. Not surprisingly, we saw increases in the statistical significance of these metabolic changes when using visibly silenced leaves according to the bleached phenotype, rather than simply harvesting the leaves that emerge postinfection (Figure 6, A–D).

## Discussion

Here we report an improved VIGS methodology that uses a “double knockdown” approach, in which both the gene of interest and a marker gene allow selective harvesting of tissue that has actually been affected by silencing. A transgenic line of *Arabidopsis thaliana* expressing GFP has also been subjected to an alternative double knockdown approach using the transgenic GFP as a marker (Burch-Smith et al., 2006). However, knockdown in a wild-type plant is a more versatile approach, and our analysis indicates that while silencing of the endogenous PDS gene does cause some minor disruptions in the baseline plant metabolism, the chemotype of silenced biosynthetic genes can be readily observed.

The development of the marker-based VIGS screening system described here enabled us to more accurately and reliably probe the function of genes. Using this VIGS method, we discovered that transcript CRO_T111318 encodes the enzyme SS (CrSS; Figures 3, 4). Silencing data of CrSS using the standard pTRV2 vector compared with data using the marker containing pTRV2-PDS vector clearly show that this modified silencing method gives data with improved statistical parameters (Figure 6).

**Figure 7:**
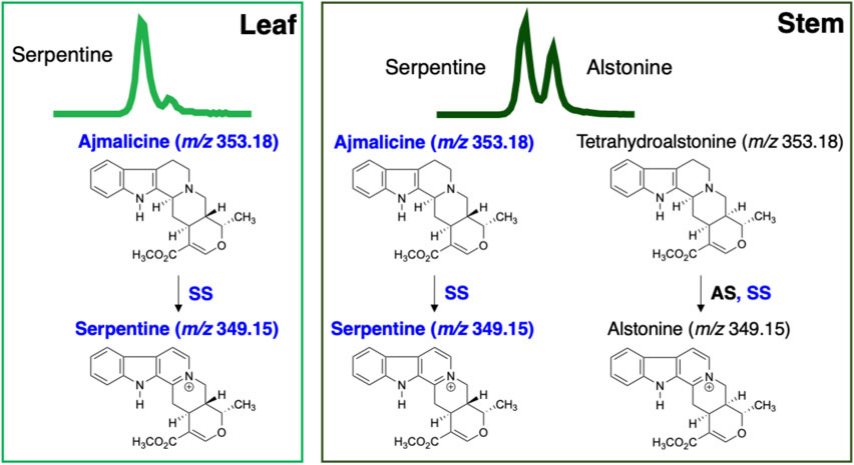
Proposed model of serpentine biosynthetic pathway in planta. More serpentine than alstonine accumulates in leaf tissues (light green chromatogram) compared to stem (dark green chromatogram). This is consistent with both the substrate specificity of CrSS (highlighted by blue font) and the gene expression profiles of CrSS and CrAS.

CrSS is a homolog (69% amino acid sequence identity) of the cytochrome P450 AS that produces the stereoisomer of serpentine, alstonine (Dang et al., 2018). Previous biochemical studies suggested that CrSS would be a vacuole-localized peroxidase; it was reported that the serpentine precursor ajmalicine was taken up into isolated *C. roseus* vacuoles in vitro, and then converted into serpentine by the basic peroxidases present in this organelle (Blom et al., 1991). Epifluorescence microscopy reported here suggests instead that the enzyme CrSS is an ER-localized cytochrome P450 (Supplemental Figure S11), similar to other TIA-related CYP450s (Besseau et al., 2013; Carqueijeiro et al., 2018; Dang et al., 2018), indicating that serpentine is made on the ER, not the vacuole. Thus, it is serpentine, not the substrate ajmalicine, that must be imported into the vacuole, the location of β-carboline alkaloid accumulation (Blom et al., 1991). Heterologous expression of CrSS in yeast shows that the enzyme can also convert THA to alstonine in addition to converting ajmalicine to serpentine (Figure 5). Overall, the pattern of serpentine and alstonine accumulation in tissues appears to be consistent with the gene expression pattern of CrAS and CrSS (Supplemental Figure S2). Previously reported single-cell metabolomic analysis showed that ajmalicine and serpentine localize to idioblast cell and laticifer cell (Figure 1; Yamamoto et al., 2016; Yamamoto et al., 2019), suggesting that serpentine would be produced in both specialized cells. It is possible that CrSS may be multifunctional in planta, contributing to production of both alstonine and serpentine in plant tissues (Figure 7).

The pTRV2 is the most widely used VIGS vector used in plant biology, and the marker PDS can be used to check silencing in all chlorophyll containing tissues. Thus, we anticipate that this method can be used in a wide variety of plant systems. Although this method will not improve the efficacy of VIGS in plant systems recalcitrant to transformation, we anticipate that this method will greatly improve interpretation of silencing data in plants with low silencing efficiencies.

## Materials and methods

### Plant growth conditions

*Catharanthus roseus* cv “Little Bright Eyes” seeds were obtained from B and T World Seeds (2011) and propagated in house. *Catharanthus roseus* cv “Sunstorm Apricot” seeds were obtained from Syngenta (2012). *Catharanthus roseus* plants were cultured at 21°C–28°C in 16-h/8-h light/dark. We produced second-generation plants (Sunstorm Apricot 1–2) from the seeds of Sunstorm Apricot. We used Little Bright Eyes and Sunstorm Apricot for making cDNA for cloning and Sunstorm Apricot 1–2 for VIGS experiments.

### Chemicals

Commercially available TIA standards, ajmalicine (41111, Sigma-Aldrich), serpentine hydrogen tartrate (SRP01300s, Sequoia Research Products Ltd), THA (R289094, Sigma-Aldrich), and ajmaline (448000, Toronto Research Chemicals) were used for annotation peak or normalization. Alstonine was synthesized as previously described (Dang et al., 2018).

### Microscopy conditions

*Catharanthus roseus* leaves and serpentine were observed with Stereomicroscopy (M205FA, Leica). The set of ET UV LP (excitation: 325–375 nm, emission: LP420 nm) and LED Lightsource Sola 365 (Lumencor, Inc) was used for excitation of *C. roseus* leaves, ajmalicine (1 ppm) and serpentine hydrogen tartrate (1 ppm; Figure 1).

### Cloning and constructs

VIGS vectors, pTRV1 and pTRV2-MCS, were obtained from The Arabidopsis Biological Resource Center (https://www.arabidopsis.org). *Catharanthus roseus* RNA was extracted from the first pair leaf of Little Bright Eyes or Sunstorm Apricot (6 weeks old) to make cDNA for cloning of gene fragments. Fragments of open reading frames encoding *C. roseus* PDS (460 bp) were amplified with Platinum SuperFi DNA Polymerase (Thermo Fisher Scientific). The PDS marker gene was inserted in pTRV2-MCS vector with ECORI and NCOI sites with restriction cloning (Supplemental Table S1). The target genes (CS and three candidate genes) were similarly amplified from *C. roseus* cDNA and were inserted in pTRV2-PDS-MCS vector at BamHI and XhoI sites with In-fusion HD Cloning Kit (639650, Takara Clontech; Supplemental Table S1). The plasmid was transformed into *Escherichia coli* (636766, Stellar Competent Cells, Takara Clontech). The resulting vector was purified using Wizard Miniprep kit (Promega) and verified by sequencing, using pTRV2 vector-specific primers (Supplemental Table S1).

The three candidate genes were identified by performing a BLAST analysis of the publicly available *C. roseus* transcriptome from the Medicinal Plant Genomics Resource (MPGR) consortium (medicinalplantgenomics.msu.edu) using AS as a query (Kellner et al., 2015). The BLAST hits were analyzed by phylogenetic analysis. Phylogenetic tree was made by using the Geneious Tree Builder program in the Geneious software package (Version 2020. 1. 2).

### *Agrobacterium tumefaciens* infection of *C. roseus*

*Agrobacterium tumefaciens* GV3101 was used for all VIGS experiments. Electrocompetent *A. tumefaciens* was transformed with pTRV1 or one of the pTRV2 vectors described above. Transformants were screened by PCR with the pTRV2 vector-specific primers to ensure that the cultures contained the expected construct (Supplemental Table S1). Positive transformants were used to inoculate 10 mL LB containing 50 μg mL^−1^ kanamycin, 25 μg mL^−1^ gentamicin, and 100 μg mL^−1^ rifampin, which were grown for 48 h at 28°C. These cultures were pelleted at 5,000*g* and resuspended in infiltration solution (10 mM MES, 100 μM acetosyringone, 10 mM MgCl_2_). Strains harboring pTRV2 constructs were then mixed in a 1:1 ratio with strains harboring pTRV1, and this mixture was used to inoculate the plants. *Catharanthus roseus* (cv Sunstorm Apricot) 4- to 6-week-old plants were inoculated with the *A. tumefaciens* culture mix by using a pair of fine forceps with loops at the tips. The forceps were dipped in *A. tumefaciens* culture mix and then used to pinch the stem, just below the apical meristem and the youngest leaf pair. Plants used for VIGS had at least one pair of fully expanded true leaves. Four-weeks post infection bleached leaves were harvested for analysis. Then, the metabolites were extracted with MeOH including 2 ppm ajmaline, after leaf samples were frozen in liquid nitrogen and pulverized with a tissue grinder. VIGS experiments were conducted with four to five biological replicates for initial screening experiments. Figures shown were created from 5 to 10 biological replicates data.

### Expression of recombinant CrSS in *S. cerevisiae*

The coding sequence of CrSS was amplified using SuperFi II polymerase (ThermoFischer) using the primers listed in Supplemental Table S1 from leaf *C. roseus* cDNA. The amplicon was cloned into the multiple cloning site of pESC-Leu2d:AaCPR (Ro et al., 2008; Dang et al., 2018) using the In-fusion HD Cloning Kit (639650, Takara Clontech). The resulting vector was purified using Wizard Miniprep kit (Promega) and verified by sequencing, using Gal10 primers (Supplemental Table S1). The expression vector was transformed into YPL154C:Pep4KO *S. cerevisia*e strain (Nguyen et al., 2010) and plated on selective SC-Leucine +2% (w/v) amino acid dropout media. Colony PCR using Phire Hot Start II polymerase (ThermoFischer) and Gal10 primers were used to confirm the positive transformants.

Microsomes harboring the recombinant protein were prepared using the recombinant pESC-Leu2d:AaCPR:CrSS yeast strain. Briefly, the expression strain was cultured overnight in SC-Leucine + 2% (w/v) glucose dropout media at 30°C with shaking (200 rpm). The starter culture was then diluted 1:100 in SC-Leucine + 2% (w/v) glucose medium for 16 h at 30 °C with shaking (200 rpm). The cells were harvested by centrifugation at OD_600_ = 1.0 and resuspended in SC-Leucine + 2% (w/v) galactose media to induce recombinant protein expression. The culture was incubated for 48 h at 30°C with shaking (200 rpm). The cells were harvested using centrifugation, washed in 20 mL TEK buffer (TE + 0.1 M KCl), and lysed in 10 mL TES-B buffer (TE + 0.6 M sorbitol) using an LM-20 Microfluidiser (Microfluidics) at 30,000 psi, and centrifuged at 10,000*g* for 10 min at 4 °C. The resulting supernatant was centrifuged at 110,000*g* for 70 min at 10°C. The obtained microsomal pellet was resuspended in 3 mL TEG buffer (TE + 20% (v/v) glycerol). The recombinant CrSS protein was detected by a western blot using HRP-conjugated anti-FLAG antibodies (ThermoFischer), and developed using Expedeon ECL Extreme (Expedeon) chemilluminescent substrate (Supplemental Figure S8).

### Enzyme activity assays

The activity assays of microsomal preparations of recombinant CrSS were performed by incubating 4 μL of the microsomal preparation (150–200 μg per reaction) with 30 μM of selected substrate in the presence of 100 mM HEPES-NaOH buffer (pH 7.5) and 150 μM NADPH in total 50-μL assay volume for 60 min, at 30 °C with 300 rpm shaking. The reactions were quenched with 1– assay volume of MeOH containing 2 ppm ajmaline (internal standard), and centrifuged at 16,000 g to precipitate the proteins. The resulting supernatant was subjected to UPLC–MS/MS analysis as described below. Substrate competition assays were carried out using the same method, differing only in the presence of ajmalicine and THA at equal concentrations of 30 μM of each substrate. Each enzyme assay was repeated a minimum of three times. For the assays in which substrate was fed to whole-cell yeast cultures, 30 μM (final concentration) of selected substrate was added to the culture medium.

### UPLC–MS/MS analysis of silenced leaf tissue and in vitro enzyme assays

Two UPLC methods were used for analysis. A short method that did not effectively separate the diastereomers (tetrahydroalstonine/ajmalicine and alstonine/serpentine) was used for rapid screening. A longer method was used to separate these diastereomers. Both methods used a UPLC (Elute LC, Bruker), coupled with an Impact II QToF mass spectrometer (Bruker). The mass spectrometer was operated at the following parameters: ESI+ ionization, 500 V source voltage, 4,500 V capillary voltage, 2.5 bar nebulizer gas pressure, 11 L min^−1^ drying gas flow, 250°C drying gas temperature.

For the short method, chromatography was performed on a Phenomenex Kinetex XB-C18 column (2.1 × 100 mm^2^, 2.6 μm) at a flow rate of 0.6 mL min^−1^ and 40°C column oven temperature. The column was equilibrated in solvent A (0.1% [v/v] formic acid in water) and the following gradient was applied: 0 min, 10% B (0.1% [v/v] formic acid in acetonitrile); 1–6 min, 30% B; 6.10–7.50 min, 100% B; and 7.60–9 min at 10% B for column re-equilibration. For the long method used for separation of diastereomers, chromatography was performed on a Waters Acquity BEH C18 column (2.1 × 50 mm^2^, 1.7 μm) at a flow rate of 0.3 mL min^−1^ and 60°C column oven temperature. The column was equilibrated in solvent A (0.1% [v/v] NH_4_OH in water) and the following gradient was applied: 0 min, 5% B (100% acetonitrile); 1–18 min, 65% B; 18.10–20.10 min, 100% B; and 20.60–22.60 min at 5% B for column re-equilibration. For the yeast samples, a 1-min delay to waste was used to eliminate media components on the mass spectrometer detector.

### Gene expression analysis

Tissue-specific gene expression patterns for *C. roseus* wild-type plants were generated using previously published transcriptomic data from the MPGR consortium (medicinalplantgenomics.msu.edu; Supplemental Figure S2). To assess the gene expression levels after silencing, RNA was extracted from knockdown leaf samples with RNeasy Plant Mini Kit (QIAGEN) after leaf samples were crushed with a tissue grinder (*n* = 5). Three replicates were selected for each sample (pTRV2EV, PDSEV, PDSSS, PDSCS; Supplemental Figure S4). Those RNA samples were subjected to RNA-Seq analysis using a commercial vendor (BGI, Hong Kong). The gene expression of the knockdown target genes was then assessed using the fragments per kilobase of exon per million mapped reads (FPKM) of values of the RNA-Seq data.

### Transient overexpression of CrSS in *C. roseus*

The coding sequence of CrSS was amplified with primers oxSS-F and oxSS-R (Supplemental Table S1), which introduce an AgeI restriction site in both extremities. The PCR product was digested and ligated in an AgeI/XmaI-digested pEAQ-HT plasmid in order to generate the pEAQ-HT:CrSS-6HIS construct. The plasmid was electroporated in *A. tumefaciens* GV3101, and cells were prepared for infiltrations as described in the section on VIGS methodology. Agrobacteria harboring either an EV (pEAQ-HT) or the pEAQ-HT:CrSS-6HIS (oxCrSS) vector were mixed with Agrobacteria harboring a GFP-expressing vector (pEAQ-HT:GFP-6HIS) in a final OD_600_ = 0.25 each. *Catharanthus roseus* plants (cv Little Bright Eyes) were vacuum infiltrated and leaf areas exhibiting GFP fluorescence were collected at 6 d post infiltration for further analyses. Proteins were extracted as described previously (Carqueijeiro *et al.*, 2020) and resolved on 8% SDS–PAGE, followed by Coomassie Brilliant Blue staining or western blotting. Western blot analysis was performed with an anti-HIS tag primary antibody (#ab213204, Abcam) and a horseradish peroxidase-conjugated secondary antibody (#1706515, Bio-Rad) following standard protocols. The alkaloid content of agroinfiltrated leaves was determined using UPLC-MS analysis as described previously (Dugé de Bernonville et al., 2017).

### Subcellular localization studies

In silico prediction of the subcellular localization of CrSS was performed by submitting the amino acid sequence to DeepLoc (http://www.cbs.dtu.dk/services/DeepLoc) and LocTree3 (www.rostlab.org/services/loctree3) servers. Prediction of transmembrane helices was performed by submitting the amino acid sequence of CrSS to the TMHMM v2 server (www.cbs.dtu.dk/services/TMHMM). The coding sequence of CrSS was amplified with primers SSYFP-F and SSYFP-R (Supplemental Table S1), which introduce a SpeI restriction site in both extremities. The PCR product was digested and cloned in a SpeI-digested pSCA-YFP plasmid to generate the expression cassette of YFP fused in frame to the C-terminus of CrSS (CrSS-YFP) and to maintain the functionality of the predicted N-terminal transmembrane helix. *Catharanthus roseus* cells were transiently cotransformed with CrSS-YFP and an ER-CFP marker (Nelson *et al.*, 2007). Particle bombardment and epifluorescence microscopy were performed as described previously (Carqueijeiro *et al.*, 2018). Briefly, *C. roseus*-plated cells were bombarded with DNA-coated gold particles (1 mm) and 1,100 psi rupture disk at a stopping-screen-to-target distance of 6 cm, using the Bio-Rad PDS1000/He system. Cells were cultivated for 16–38 h before being harvested and observed. The subcellular localization was determined using an Olympus BX-51 epifluorescence microscope equipped with an Olympus DP-71digital camera and a combination a YFP filter set (Chroma#31040, 500–520 nm excitation filter, 540–580 nm emission filter) and a Cyan GFP filter set (Chroma#31044v2, 426–446 excitation filter, 460–500 nm bandpass emission filter), respectively. YFP and CFP fluorescence are successively acquired (exposure time 50 ms) and merged with the CellD imaging software while the morphology of transformed cells is observed with differential interference contrast.

### Accession numbers

*Catharanthus roseus* SS is GenBank MT829151. CrAS is GenBank W8JDE2.1.

## Supplemental data

The following materials are available in the online version of this article. 

**Supplemental Figure S1**. VIGS treated plants 4- to 5-weeks postinfection.

**Supplemental Figure S2**. Metabolome and transcriptome data of different tissues of wild-type *C. roseus*.

**Supplemental Figure S3**. The effect of silencing CrCS and CrSS on metabolites.

**Supplemental Figure S4**. Gene expression levels after knockdown.

**Supplemental Figure S5**. Phylogenetic tree of alstonine synthase homologues.

**Supplemental Figure S6**. LC–MS analysis of *C. roseus* leaves with silenced SS.

**Supplemental Figure S7**. In vitro assay of SS using yeast microsomes.

**Supplemental Figure S8**. Expression of CrSS in *S. cerevisiae*.

**Supplemental Figure S9**. Transient overexpression of CrSS in *C. roseus*.

**Supplemental Figure S10**. Detection of CrSS in transiently transformed *C. roseus*.

**Supplemental Figure S11**. Subcellular localization of CrSS.

**Supplemental Table S1**. Primer sequences, vectors and restriction sites used in this study.

## Supplementary Material

kiab285_Supplementary_Data

## Notes

K.Y. and S.E.O. planned and coordinated the project and wrote the manuscript. K.Y. designed the research. K.Y. performed making constructs and VIGS analysis. K.Y. and D.G. performed in vitro assay for serpentine synthase. K.K., E.A.S., and V.C. performed transient overexpression and subcellular localization studies. T.M. and L.C. helped to prepare the manuscript and to analyze data.

The author responsible for distribution of materials integral to the findings presented in this article in accordance with the policy described in the Instructions for Authors (https://academic.oup.com/plphys/pages/general-instructions) is: Sarah E. O’Connor (oconnor@ice.mpg.de).
