# Supplementary material for: Improved virus-induced gene silencing allows discovery of a serpentine synthase gene in Catharanthus roseus
Source: Plant Physiol. 2021 Jun 19;187(2):846–57. doi: 10.1093/plphys/kiab285 (PMC8491018; doi:10.1093/plphys/kiab285)
Supplement: kiab285_Supplementary_Data [file kiab285_supplementary_data.pdf]

Supplemental Information for

Improved virus-induced gene silencing allows discovery of a serpentine synthase gene in *Catharanthus roseus*

Kotaro Yamamoto[1, 2], Dagny Grzech[1], Konstantinos Koudounas[3], Emily Armor Stander[3], Lorenzo Caputi[1], Tetsuro Mimura[4], Vincent Courdavault[3], Sarah E. O'Connor[1]

[1]Department of Natural Product Biosynthesis, Max Planck Institute for Chemical Ecology, Jena 07745, Germany

[2]Graduate School of Pharmaceutical Sciences, Chiba University, Chiba 263-8522, Japan

[3]EA2106 "Biomolécules et Biotechnologies Végétales", Université de Tours, Tours 37200, France

[4]Department of Biology, Graduate School of Science, Kobe University, Kobe, Hyogo 657-8501, Japan

Corresponding author:

Sarah E. O'Connor

Email: oconnor@ice.mpg.de

Supplemental Figure S1. VIGS treated plants 4- to 5-weeks postinfection.

Supplemental Figure S2. Metabolome and transcriptome data of different tissues of wild-type *C. roseus*.

Supplemental Figure S3. The effect of silencing CrCS and CrSS on metabolites.

Supplemental Figure S4. Gene expression levels after knockdown.

Supplemental Figure S5. Phylogenetic tree of alstonine synthase homologues.

Supplemental Figure S6. LC-MS analysis of *C. roseus* leaves with silenced SS.

Supplemental Figure S7. In vitro assay of SS using yeast microsomes.

Supplemental Figure S8. Expression of CrSS in *S. cerevisiae*.

Supplemental Figure S9. Transient overexpression of CrSS in *C. roseus*.

Supplemental Figure S10. Detection of CrSS in transiently transformed *C. roseus*.

Supplemental Figure S11. Subcellular localization of CrSS.

Supplemental Table S1. Primer sequences, vectors and restriction sites used in this study.

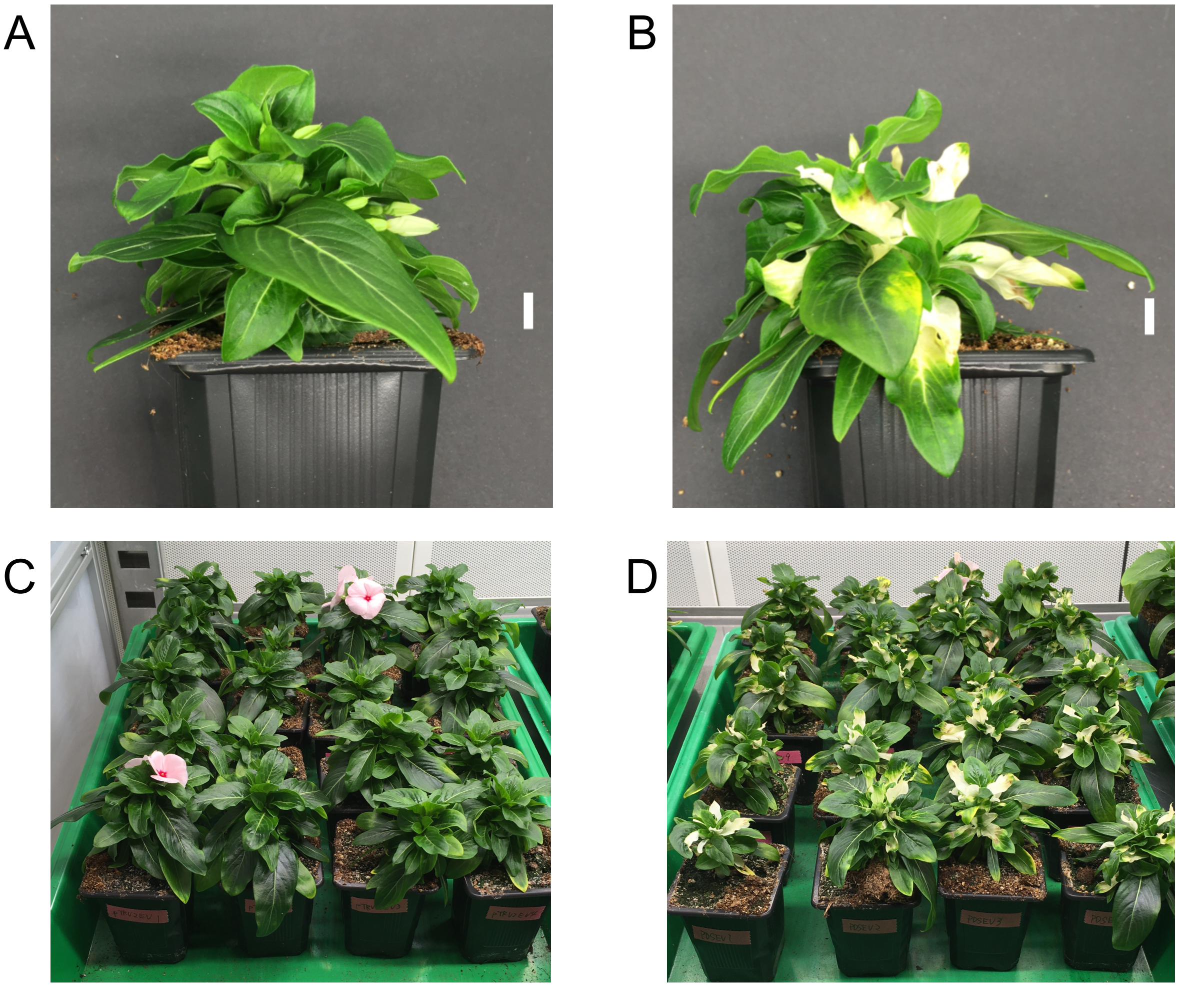

Supplemental Figure S1. VIGS treated plants 4- to 5-weeks postinfection. (A) Plant infiltrated with pTRV2EV. (B) Plant infiltrated with PDSEV (phytoene desaturase + empty vector). (C) Plants infiltrated with SS (serpentine synthase) knockdown without marker (back) and pTRV2EV (front). (D) Plants infiltrated with PDSSS (phytoene desaturase + serpentine synthase) knockdown (back) and PDSEV knockdown (front). (Scale bar = 1 cm).

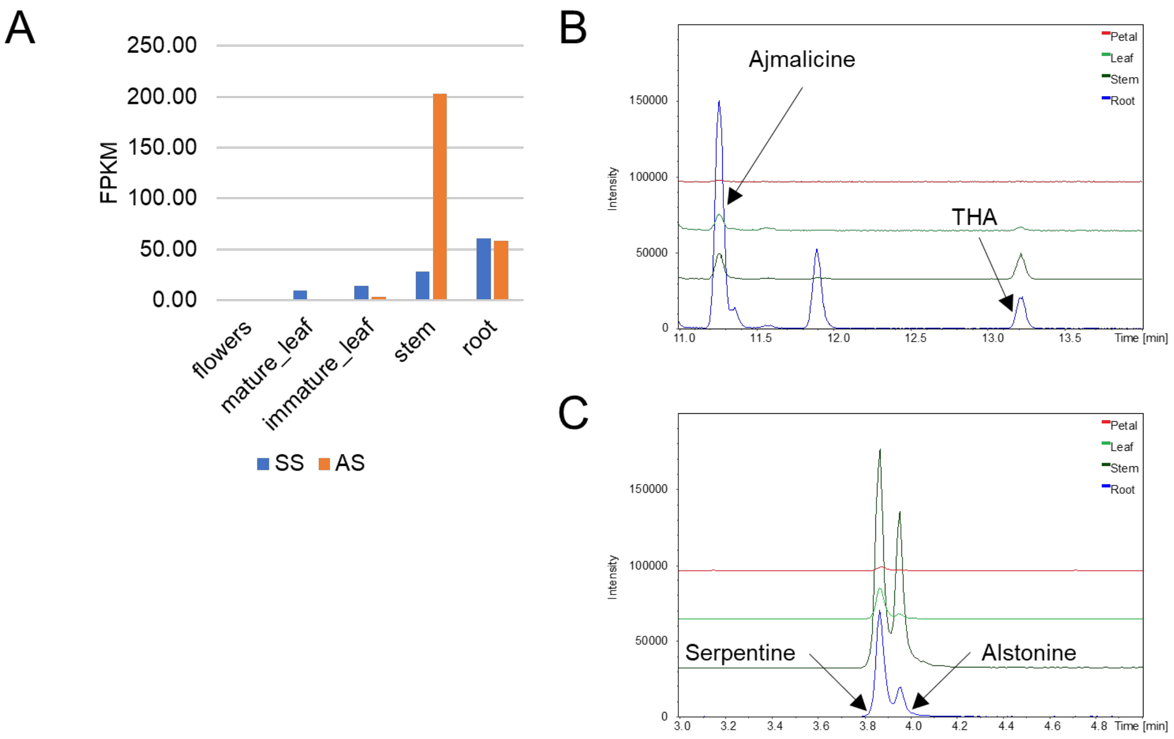

Supplemental Figure S2. Metabolome and transcriptome data of different tissues of wild-type *C. roseus*. (A) Gene expression pattern of CrSS (serpentine synthase) and CrAS (alstonine synthase) with MPGR (Medicinal Plant Genomics Resource, http://medicinalplantgenomics.msu.edu) RNA-seq datasets (cultivar name Little Bright Eyes). (B) Metabolic profile of *m/z* 353.18 (ajmalicine and tetrahydroalstonine (THA)). (C) Metabolic profile of *m/z* 349.15 (serpentine and alstonine). The amount of MeOH for extraction was calculated and normalized with the fresh weight of each sample for making LC-MS chromatogram.

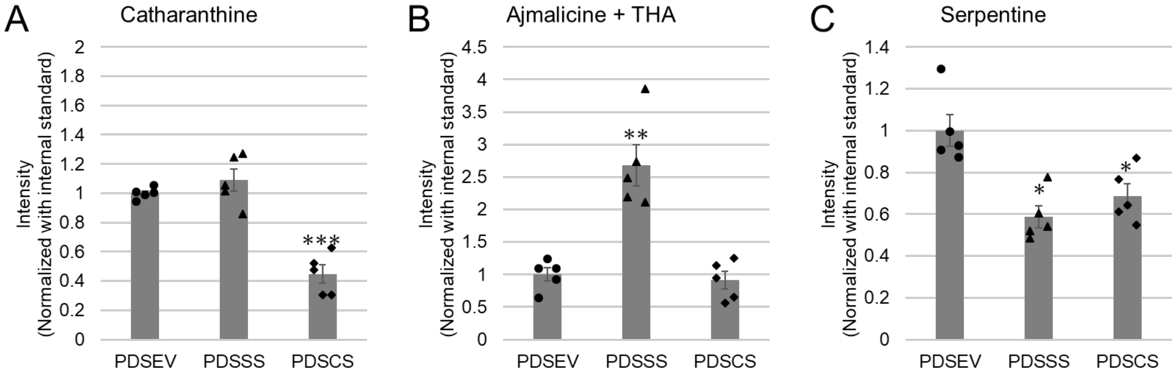

Supplemental Figure S3. The effect of silencing CrCS and CrSS on metabolites. Silencing of the control gene, catharanthine synthase (PDSCS), on selected metabolites is compared to the effect of silencing serpentine synthase (CrSS, CRO_T111318; characterization reported in Supplemental Figures S6-8). Catharanthine synthase and serpentine synthase are biosynthetic enzymes from two unrelated terpenoid indole alkaloid metabolic pathways. (A) Catharanthine, the expected product of catharanthine synthase, is reduced when the plant is infiltrated with PDSCS. In contrast, catharanthine levels are unaffected when serpentine synthase is silenced. (B) Ajmalicine + Tetrahydroalstonine (THA) levels increase when serpentine synthase is silenced, which is expected, since ajmalicine is the substrate of serpentine synthase. As expected, these metabolite levels are not perturbed when catharanthine synthase is silenced. (C) Serpentine levels are, not surprisingly, reduced when the plant is infiltrated with the serpentine synthase silencing construct. For all panels, the y-axis represents the intensity level in arbitrary units that was calculated and normalized, with the control value normalized as 1 ($n = 5$). For all panels, error bar shows SE (standard error). For all panels, student t-test was used for statistical analysis: *$p<0.05$, **$p<0.01$, ***$p<0.005$.

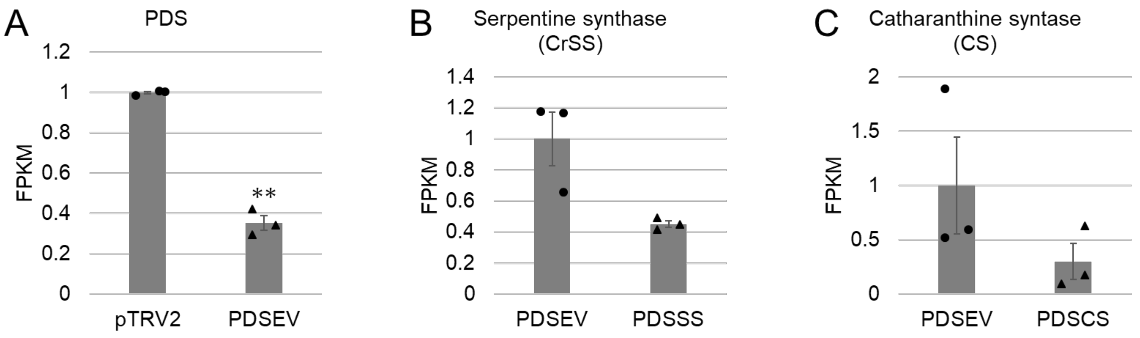

Supplemental Figure S4. Gene expression levels after knockdown. (A) PDS (phytoene desaturase) gene expression level after knockdown of phytoene desaturase (PDSEV) compared to pTRV2 empty vector (pTRV2EV). (B) Serpentine synthase (CRO_T111318) gene (characterization reported in Supplemental Figures S6-8) expression level after knockdown with PDS (PDSSS) compared to PDS vector (PDSEV). (C) CS (catharanthine synthase) gene expression level after catharanthine synthase knockdown with PDS (PDSCS) compared to PDS vector (PDSEV). For all panels, the gene expression level (y axis) was calculated and normalized (control value was normalized as 1) with FPKM (Fragments Per Kilobase of transcript per Million mapped reads) of knockdown samples (n = 3). Error bar shows SE for all panels. Student t-test was used for statistical analysis: **p<0.01 for all panels.

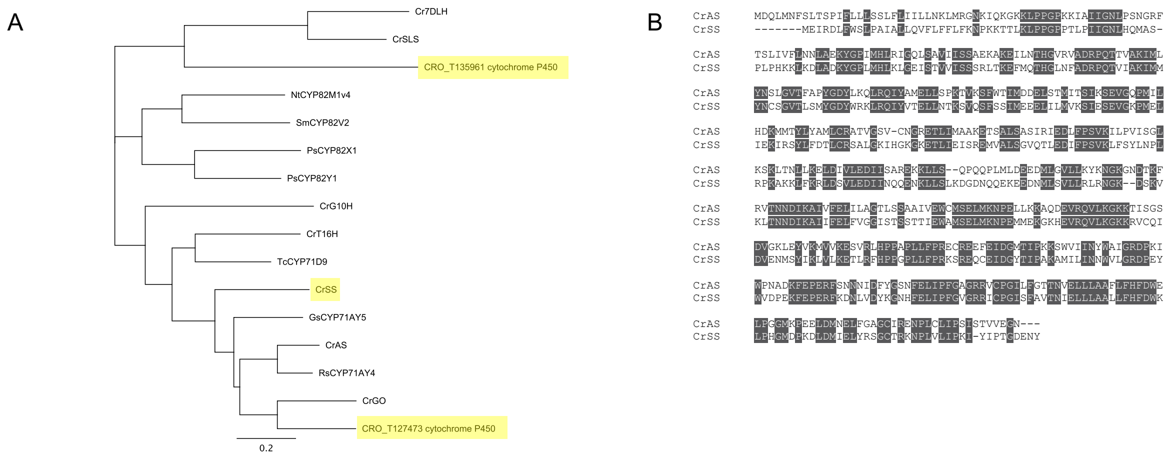

Supplemental Figure S5. Phylogenetic tree of alstonine synthase homologues. (A) CrSS, along with the other two gene candidates that were silenced, are highlighted in yellow. (B) Amino acid sequence alignment of CrAS and CrSS (CRO_T111318). The two proteins share 69% amino acid sequence identity. CrT16H, *Catharanthus roseus* tabersonine 16-hydroxylase; CrG10H, *C. roseus* geraniol 10-hydroxylase; Cr7DLH, *C. roseus* 7-deoxyloganic acid hydroxylase; CrSLS, *C. roseus* secologanin synthase; TcCYP71D9, *Theobroma cacao* cytochrome CYP71D9; SmCYP82V2, *Salvia miltiorrhiza* cytochrome P450 CYP82V2; RsCYP71AY4, *Rauvolfia serpentina* CYP71AY4; PsCYP82Y1, *Papaver somniferum* cytochrome P450 CYP82Y1; PsCYP82X1, *Papaver somniferum* cytochrome P450 CYP82X1; NtCYP82M1v4, *Nicotiana tabacum* CYP82M1v4; GsCYP71AY5, *Gelsemium sempervirens* CYP71AY5. Scale bar indicates number of amino acid substitutions per site.

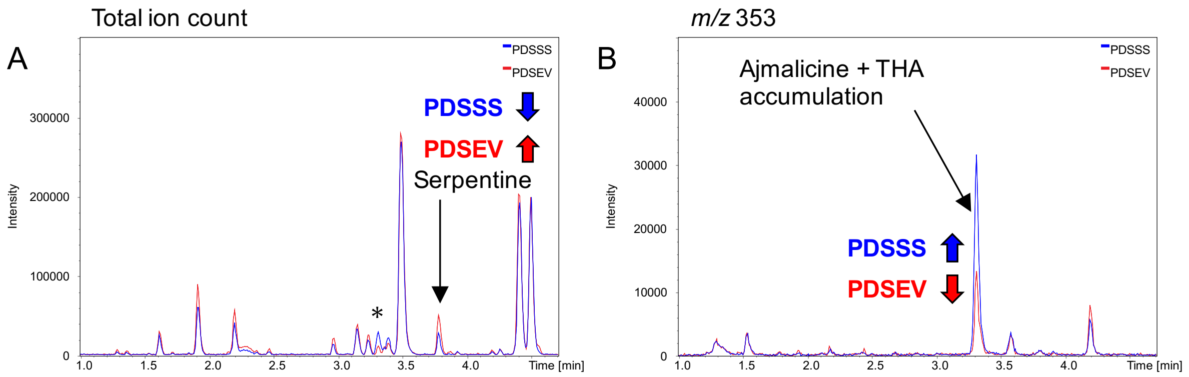

Supplementary Figure S6. LC-MS analysis of *C. roseus* leaves with silenced SS. (A) Total ion chromatogram of silenced leaves (blue) and leaves transformed with empty vector constructs (red). Serpentine decreases in response to silencing. The peak corresponding to ajmalicine and tetrahydroalstonine is marked with an asterisk (*m/z* 353.18). (B) Chromatogram of silenced (blue) and empty vector (red) leaves (*m/z* 353.18). Ajmalicine and tetrahydroalstonine (THA) increases substantially in the serpentine synthase silenced (PDSSS) leaves. Y axis represents ion signal intensity in arbitrary units.

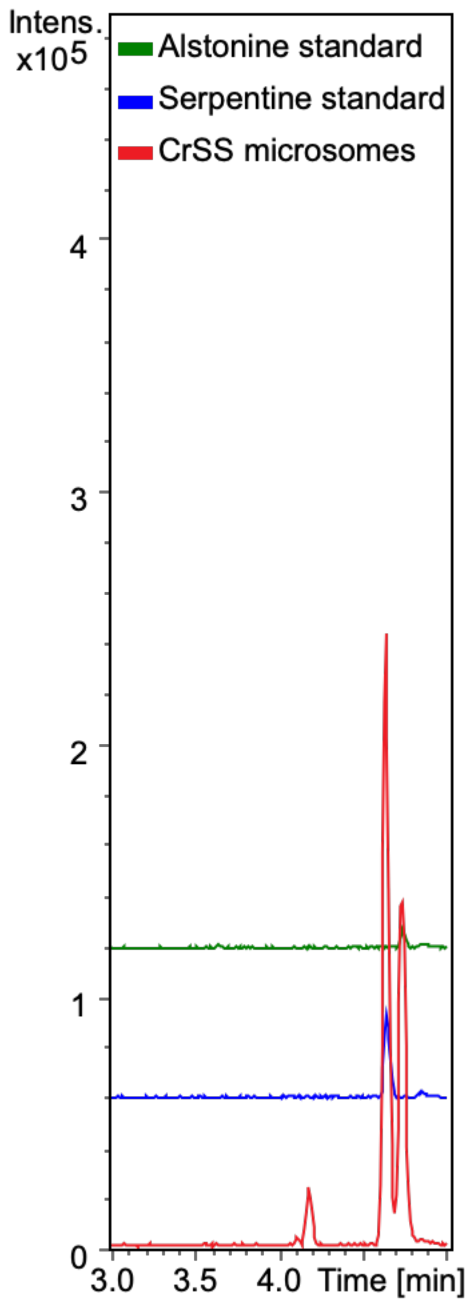

Supplemental Figure S7. In vitro assay of SS using yeast microsomes. Chromatogram displaying results of an in vitro microsomal CrSS (serpentine synthase) protein substrate competition assay containing 30 µM of both ajmalicine and tetrahydroalstonine for 60 minutes. CrSS produces both serpentine and alstonine under these conditions. Experiments were repeated in triplicate. Y axis represents ion signal intensity in arbitrary units.

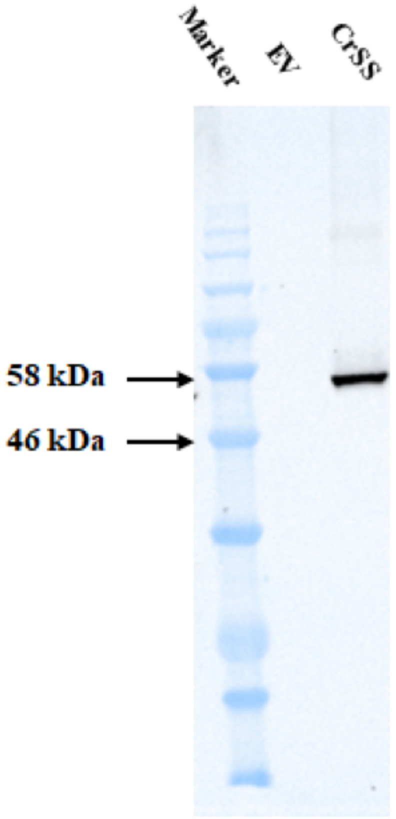

Supplemental Figure S8. Expression of CrSS in *S. cerevisiae*. The protein was detected by Western blot in yeast microsomal preparations using an anti-FLAG antibody.

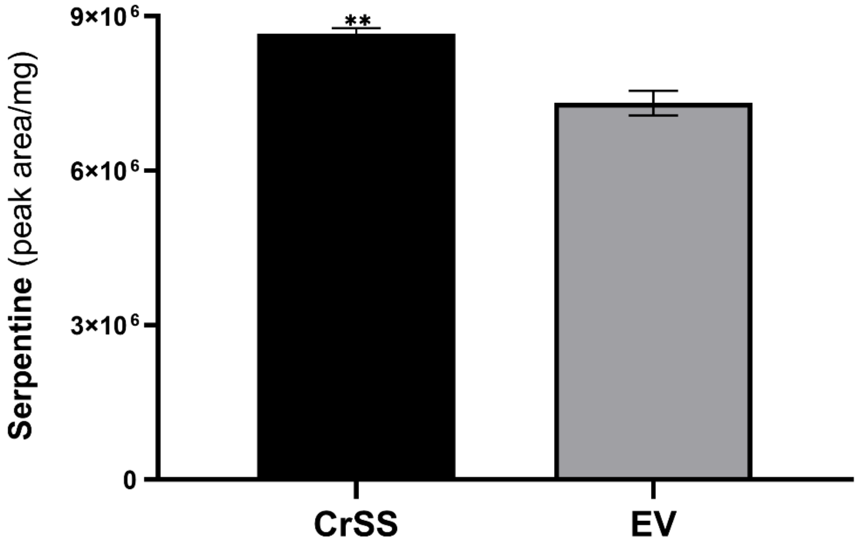

Supplemental Figure S9. Transient overexpression of CrSS in *C. roseus*. Serpentine accumulation (peak area per mg of dry weight) in *C. roseus* plants infiltrated with pEAQ-HT:GFP-6HIS and either pEAQ-HT:CrSS-6HIS (CrSS) construct or empty vector (EV) at 6 dpi. Two technical replicates of four biological replicates were analyzed. (Mean ± SE; ** $p \leq 0.01$; Student's t-test).

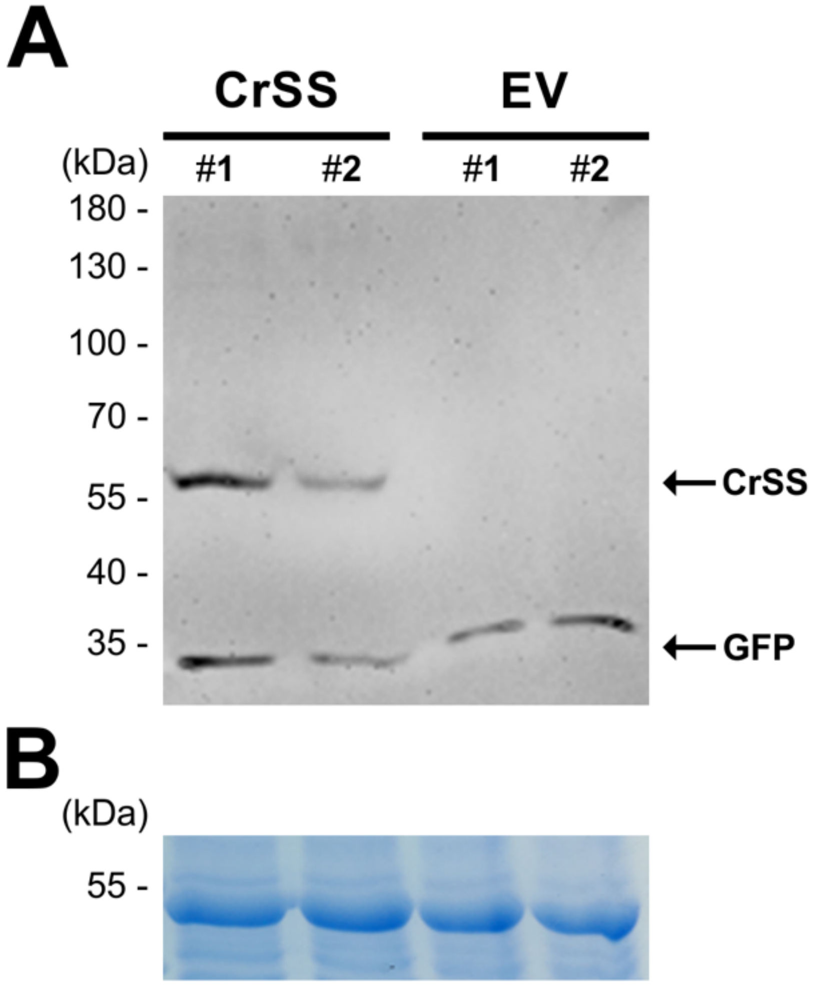

Supplemental Figure S10. Detection of CrSS in transiently transformed *C. roseus*. (A) Western blot and (B) CBB staining of crude protein extracts (20 µg) from *C. roseus* plants infiltrated with pEAQ-HT:GFP-6His and either pEAQ-HT:CrSS-6His (CrSS) construct or empty vector (EV) at 6 dpi. Numbers above indicate biological replicates and numbers on the left indicate molecular weights.

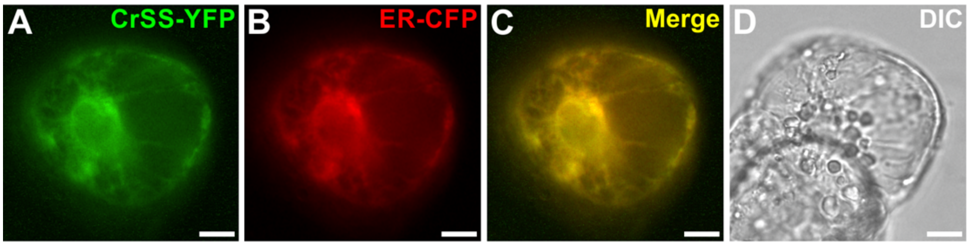

Supplemental Figure S11. Subcellular localization of CrSS. *C. roseus* cells were transiently transformed with (A) the CrSS-YFP construct and (B) an ER-CFP marker. (C) Co-localization was confirmed after merging the two fluorescence signals. (D) Cell morphology was observed with differential interference contrast (DIC). Bars = 10 µm.

Supplemental Table S1. Primer sequences, vectors and restriction sites used in this study.

Primer	Vector	Restriction site	Sequence
PDS-EcoRI-F	pTRV2 vector	EcoRI	CGAGAATTCAGGTTTGGGGGGTTTGTG
PDS-NcoI-R	pTRV2 vector	NcoI	CGACCATGGTACGCCTTGCTTTCTCATCC
PDS-SS-F	pTRV2PDS vector	BamHI	CGTACCATGGGGATCAAAAATAAGGCTATCTTCGTAAGGTGAAG
PDS-SS-R	pTRV2PDS vector	XhoI	ATGCCCGGGCCTCGACTATTATGGTTAAATTGGTGGCCTTTG
SS-F	pTRV2 vector	BamHI	GCCTCCATGGGGATCAAAAATAAGGCTATCTTCGTAAGGTGAAG
SS-R	pTRV2 vector	XhoI	ATGCCCGGGCCTCGACTATTATGGTTAAATTGGTGGCCTTTG
PDS-CS-F	pTRV2PDS vector	BamHI	CGTACCATGGGGATCTAATATTCATCTTTGTTTTACGTTCTTACTTTC
PDS-CS-R	pTRV2PDS vector	XhoI	ATGCCCGGGCCTCGACGCATTATTCAAATTTTTTACTTATCTTCTC
pESC-pOPINsite-CrSS-F	pESC-Leu2d::AaCPR vector	SpeI	ACCCTCACTAAAGGGCGGCCGCAACCATGGAGATCCGAGACCTCTT
pESC-pOPINsite-CrSS-R	pESC-Leu2d::AaCPR vector	SpeI	GTCATCCTTGTAATCCATCGATACATAATTTTCATCTCCAGTGGGAATATAAATT
PDS-CRO_T127473-F	pTRV2PDS vector	BamHI	CGTACCATGGGGATCCAAAAGGGACACCGACAGC
PDS-CRO_T127473-R	pTRV2PDS vector	XhoI	ATGCCCGGGCCTCGAACATGTACAAAATCTAAACATTCCTAACTC
PDS-CRO_T135961-F	pTRV2PDS vector	BamHI	CGTACCATGGGGATCGGAATGAAAAATGGTGTTATCTATAGTTTG
PDS-CRO_T135961-R	pTRV2PDS vector	XhoI	ATGCCCGGGCCTCGATTAAAGACTTCTATAACTCTTTTTGGAGAG
pTRV2-F	pTRV2 vector (Colony PCR)		GATGGACATTGTTACTCAAGGAAGC
pTRV2-R	pTRV2 vector (Colony PCR)		CAGTCGAGAATGTCAATCTCGTAGG
Gal10-primer-F	pESC vector (Colony PCR)		GGTGGTAATGCCATGTAATATG
Gal10-primer-R	pESC vector (Colony PCR)		GCAAGGTAGACAAGCCGACAAC
oxSS-F	pEAQ-HT vector	AgeI	CTGAGAACCGGTATGGAGATCCGAGACCTCTTCTGG
oxSS-R	pEAQ-HT vector	AgeI	CTGAGAACCGGTATAATTTTCATCTCCAGTGGGAATATAAATTTTG
SSYFP-F	pSCA-YFP vector	SpeI	CTGAGAACTAGTATGGAGATCCGAGACCTCTTCTGG
SSYFP-R	pSCA-YFP vector	SpeI	CTGAGAACTAGTATAATTTTCATCTCCAGTGGGAATATAAATTTTG
